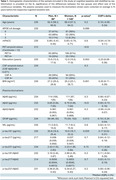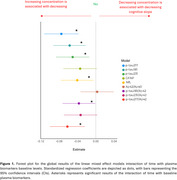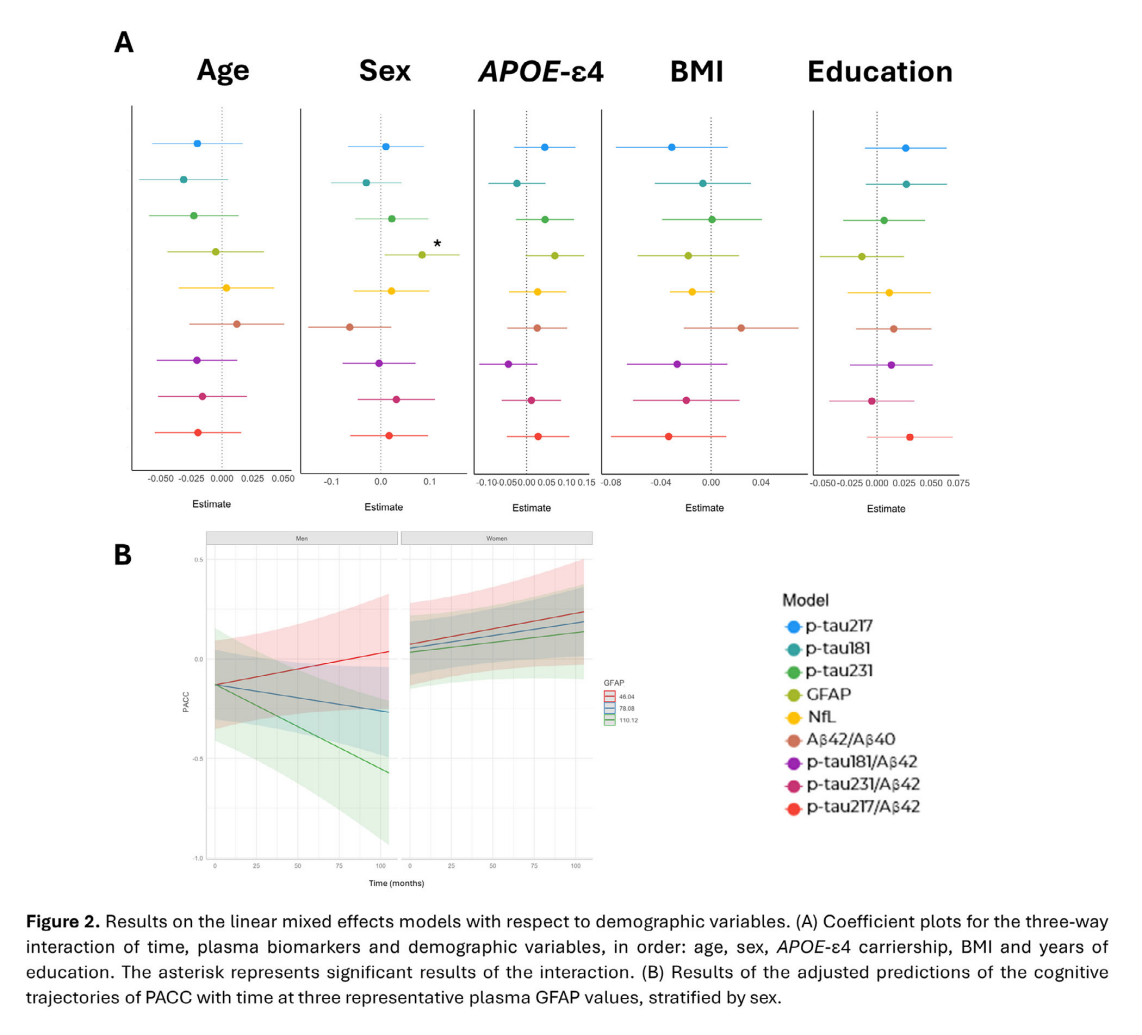# Plasma biomarkers predict long‐term longitudinal cognitive decline in individuals at‐risk for Alzheimer's disease: differences across demographic groups

**DOI:** 10.1002/alz70856_106366

**Published:** 2026-01-07

**Authors:** Armand González Escalante, Paula Ortiz‐Romero, Javier Torres‐Torronteras, Esther Jiménez‐Moyano, Helena Blasco‐Forniés, Marina De Diego‐Osaba, Federica Anastasi, Gallen Triana‐Baltzer, Hartmuth Christian Kolb, Eugeen Vanmechelen, David López‐Martos, Gonzalo Sánchez‐Benavides, Oriol Grau‐Rivera, Carolina Minguillon, Karine Fauria, Kaj Blennow, Henrik Zetterberg, Nicholas J. Ashton, Natalia Vilor‐Tejedor, Marta del Campo, Marc Suárez‐Calvet

**Affiliations:** ^1^ Barcelonaβeta Brain Research Center (BBRC), Pasqual Maragall Foundation, Barcelona, Spain; ^2^ Universitat Pompeu Fabra, Barcelona, Spain; ^3^ Hospital del Mar Research Institute (IMIM), Barcelona, Spain; ^4^ Centre for Genomic Regulation (CRG), Barcelona Institute of Science and Technology (BIST), Barcelona, Spain; ^5^ Neuroscience Biomarkers, Johnson & Johnson Innovative Medicine, La Jolla, CA, USA; ^6^ ADx NeuroSciences NV, Ghent, Belgium; ^7^ Centro de Investigación Biomédica en Red de Fragilidad y Envejecimiento Saludable (CIBERFES), Instituto de Salud Carlos III, Barcelona, Spain; ^8^ Servei de Neurologia, Hospital del Mar, Barcelona, Spain; ^9^ Centro de Investigación Biomédica en Red de Fragilidad y Envejecimiento Saludable (CIBERFES), Madrid, Spain; ^10^ Centro de Investigación Biomédica en Red de Fragilidad y Envejecimiento Saludable (CIBERFES), Instituto de Salud Carlos III, Madrid, Spain; ^11^ Paris Brain Institute, ICM, Pitié‐Salpêtrière Hospital, Sorbonne Université, Paris, France; ^12^ Neurodegenerative Disorder Research Center, Division of Life Sciences and Medicine, Institute on Aging and Brain Disorders, University of Science and Technology of China and First Affiliated Hospital of USTC, 合肥, 安徽, China; ^13^ Department of Psychiatry and Neurochemistry, Institute of Neuroscience & Physiology, the Sahlgrenska Academy at the University of Gothenburg, Mölndal, Gothenburg, Sweden; ^14^ Clinical Neurochemistry Laboratory, Sahlgrenska University Hospital, Mölndal, Västra Götaland län, Sweden; ^15^ Department of Psychiatry and Neurochemistry, Institute of Neuroscience and Physiology, The Sahlgrenska Academy, University of Gothenburg, Mölndal, Sweden; ^16^ UK Dementia Research Institute, UCL Institute of Neurology, University College London, London, England, United Kingdom; ^17^ Hong Kong Center for Neurodegenerative Diseases (HKCeND), 香港, China; ^18^ Wisconsin Alzheimer's Disease Research Center, School of Medicine and Public Health, University of Wisconsin‐Madison, Madison, WI, USA; ^19^ Department of Neurodegenerative Disease, UCL Institute of Neurology, London, United Kingdom; ^20^ Banner Sun Health Research Institute, Sun City, AZ, USA; ^21^ Banner Alzheimer's Institute and University of Arizona, Phoenix, AZ, USA; ^22^ Institute of Psychiatry, Psychology and Neuroscience, Maurice Wohl Clinical Neuroscience Institute, King's College London, London, United Kingdom; ^23^ Department of Psychiatry and Neurochemistry, Institute of Neuroscience & Physiology, the Sahlgrenska Academy at the University of Gothenburg, Mölndal, Sweden; ^24^ Hospital del Mar Research Institute, Barcelona, Spain; ^25^ Department of Genetics, Radboud Medical University Center, Nijmegen, Netherlands

## Abstract

**Background:**

Plasma biomarkers are promising tools for detecting Ab pathology in cognitively unimpaired (CU) individuals. However, their association with cognitive trajectories and how this associations vary across demographic subgroups remain unclear. This study investigates a range of plasma biomarkers to determine whether baseline levels predict cognitive trajectories in CU individuals at‐risk of Alzheimer's disease (AD) and explores differences in these associations across age, sex, Body Mass Index (BMI), *APOE*‐ε4 carriership and years of education, regardless of amyloid status.

**Method:**

We included 235 CU participants at‐risk of AD from the ALFA+ study, who are visited every 3 years, and had cognitive trajectories assessed using a sensitive modified preclinical Alzheimer's cognitive composite (mPACC) over three visits (follow‐up: 6.56±0.52 years; Table 1). Baseline plasma biomarkers (*p*‐tau181 [Quanterix Advantage V2.1], *p*‐tau217 [Janssen], *p*‐tau231 [in‐house], Aβ42/Aβ40 [Quanterix N4PE], GFAP [N4PE], NfL [N4PE] and all *p*‐tau/Aβ42 ratios) were obtained ∼3.75 years before the first cognitive visit. Associations between these biomarkers and cognitive trajectories were analysed using linear mixed‐effects models, corrected by age, sex, BMI, years of education and time gap between biomarker and cognitive measurements. Interaction models assessed the impact of demographic characteristics (age, sex, BMI, *APOE*‐ε4 carriership and years of education) on these associations.

**Result:**

Six out of the nine plasma biomarkers were associated with mPACC changes (Figure 1), with plasma *p*‐tau fragments showing the strongest coefficients (β_p‐tau217_ = ‐0.07; β_p‐tau181_ = ‐0.06; β_p‐tau231_ = ‐0.05). Plasma GFAP showed a sex‐specific effect (Figure 2), with higher plasma GFAP being associated with mPACC decline in men only (β_GFAP_men_ = ‐0.10, *p* =  0.010). The remaining demographic variables did not show any significant interaction for any biomarker. Notably, plasma *p*‐tau/Aβ42 ratios provided no additional predictive value beyond plasma *p*‐tau biomarkers alone.

**Conclusion:**

Baseline plasma biomarkers, except for plasma Aβ42/Aβ40, predict longitudinal cognitive changes in CU individuals at‐risk of AD, with plasma *p*‐tau biomarkers exhibiting the strongest associations. Plasma GFAP exhibited sex‐specific effects, suggesting the need of personalized prediction models for specific biomarker uses. Importantly, though, most plasma biomarkers were generally robust across diverse demographic characteristics, supporting their general applicability for tracking cognitive decline in preclinical AD.